# Synergistic effects of melphalan and *Pinus kesiya* Royle ex Gordon (*Simaosong*) extracts on apoptosis induction in human cancer cells

**DOI:** 10.1186/s13020-016-0103-z

**Published:** 2016-06-29

**Authors:** Natthida Weerapreeyakul, Sasipawan Machana, Sahapat Barusrux

**Affiliations:** Faculty of Pharmaceutical Sciences, Khon Kaen University, Khon Kaen, 40002 Thailand; Graduate School, Khon Kaen University, Khon Kaen, 40002 Thailand; Faculty of Associated Medical Sciences, Khon Kaen University, Khon Kaen, 40002 Thailand

## Abstract

**Background:**

This study aims to determine the synergistic effects of the chemotherapeutic drug melphalan and the phytoconstituents extracted from *Pinus kesiya* Royle ex Gordon (*Simaosong*) in human cancer cells.

**Methods:**

*P. kesiya* twigs extracted from 50 % ethanol–water were evaluated alone (6–500 µg/mL) and in combination with melphalan (0.75–15 µg/mL). The cytotoxic effects of single extract or extract and melphalan combination were examined by a neutral red assay to investigate their antiproliferative and apoptosis induction effects in the U937 and HepG2 cell lines. Nuclei morphological change and DNA fragmentation were examined by DNA nuclei staining with 4´6-diamidino-2-phenylindole (DAPI) and agarose gel electrophoresis, respectively. The chemical constituents of the *P. kesiya* extract were assessed using gas chromatography–mass spectrometry (GC–MS) analysis. The synergistic effects of different IC_50_ ratios of the *P. kesiya* extract and melphalan combination were analyzed in each cancer cell line. The dose reduction index (DRI) was calculated to determine the extent of concentration reduction in the combination treatment compared with the concentration of each single treatment.

**Results:**

The IC_50_ ratios for melphalan to *P. kesiya* extract that caused 75 % antiproliferation could be reduced after combination. This response was greater in the U937 cells than in the HepG2 cells (all *P* < 0.001). Melphalan and *P. kesiya* extract had a similar effect on apoptosis induction both singly and in combination. *P. kesiya* extract synergized the antiproliferation and apoptosis induction effects of melphalan.

**Conclusions:**

Combining the *P. kesiya* extract with melphalan reduced toxicity while retaining the therapeutic efficacy of melphalan.

## Background

Specific and selective apoptosis induction in targeted chemotherapy is limited due to multidrug resistance and intolerably severe side effects [1, 2]. Synergistic combinations of two or more agents could overcome the toxicity and side effects associated with the high doses of chemotherapeutic drugs in monotherapy [3]. Several herbal plant extracts are rich sources of bioactive constituents that inhibit, reverse, or retard tumorigenesis [3, 4]. Additionally, some research on herbal synergistic action indicates that the whole herb produces a better effect than any single isolated active ingredient [5, 6].

The rosin of *Pinus kesiya* Royle ex Gordon (*Simaosong*) contains szemaoenin (diterpenoid) isopimaric acid, abiet-13(14)-en-8,12-epoxy-18-oic acid, abiet-8,11,13-trien-15-hydroxy-18-oic acid, pimarol, isopimarol, abiet-8,11,13-trien-18-oic acid, and 15-hydroxyabietic acid [7]. The main compounds found in *P. kesiya* turpentine are α- and β-pinene [8]. The needle oil is rich in α- and β-pinene, citronellol, bornyl acetate, β-phellandrene, camphene, and β-caryophyllene [9]. The needles of *P. kesiya* contain dichlorobenzene isomer, 1,4-cineol, α-terpinene, o-cimene and imonene enantiomers [10, 11], and monoterpenes [12].

*P. kesiya* is used to relieve flatulence, stomachache, and cough in complementary and alternative medicine [13]. The 50 % ethanol–water crude extract of the woody twigs of *P. kesiya* has an apoptotic-induction effect on both the human hepatocellular carcinoma HepG2 and the human leukemic U937 cell lines because of its cytotoxicity [14, 15]. Many studies indicate that this effect arises from the whole crude extract with its lower activity of isolated individual compounds [3–6]. The synergistic effect of the whole extract of *P. kesiya* plus the chemotherapeutic drug melphalan is of interest as it may expand the use of this herbal plant. Melphalan is an alkylating anticancer drug, leading to DNA cross-linking, DNA damage, and finally apoptosis of cancer cells [16]; however, melphalan resistance and highly toxic side effects in other tissues limit its use [17, 18]. We hypothesized that plant extracts that exhibited an anticancer activity in vitro might enhance the antiproliferative activity of melphalan, which would permit the use of a lower dosage and reduced side effects. This study aims to determine the synergistic effects of *P. kesiya* and melphalan phytoconstituents ​in human cancer cells.

## Methods

### Plant extraction

*P. kesiya* woody twigs were collected and taxonomically authenticated by Assistant Professor Thaweesak Thitimetharoach. The species of samples were determined using the Flora of Thailand [19] and the Thai Forest Bulletin [20]. The voucher (TT-OC-SK-910) was deposited at the Herbal Herbarium, Faculty of Pharmaceutical Sciences, Khon Kaen University, Khon Kaen Province, Thailand. The 50 % ethanol–water extract of *P. kesiya* twig was prepared as previously reported [14, 15]. Dried plants were cut and macerated with 50 % ethanol–water (1 kg:6 L) for 7 days with occasional manual shaking. The solvent was filtered, distilled by a rotary evaporator (RV 8 V, IKA, Germany) below 40°C, and freeze-dried to obtain the crude extract. The percent yield of the 50 % ethanol–water extract of *P. kesiya* was 4.3 %. Stock solution of *P. kesiya* extract was freshly prepared in dimethyl sulfoxide (DMSO) to make 100 mg/mL stock solution and further diluted with culture media to create a working solution (10 to 500 µg/mL).

### Gas chromatography–mass spectrometry (GC–MS) analysis

GC–MS analysis was performed as described by Weerapreeyakul et al. [21] on an Agilent 6890 N gas chromatograph (Agilent Technologies, China) coupled to an Agilent 5973 N mass selective detector (Agilent Technologies, USA) to determine the extract composition for further standardization. Capillary GC analysis was performed using a DB-5 ms (3 m × 0.25 mm id, 0.25 µm) capillary column from Agilent Technologies (J&W Scientific, USA) with helium as the carrier gas. The column initially flowed at 80 °C for 6 min at a rate of 2 mL/min and an average velocity of 52 cm/s. The temperature was raised to 280 °C (at a rate of 5 °C/min) for 24 min. The total runtime was 70 min. The injector temperature was maintained at 250 °C and the injection volume was set at 2.0 µL in the splitless mode. The interface temperature was held at 280 °C. Mass spectra were scanned from m/z 50.0 to m/z 500.0 at a rate of 1.5 scans/s with a threshold of 150. The electron impact ionization energy was 70 eV. The chemical components of the crude extracts were identified from the chromatograms and mass spectra using the Wiley 7 N.l database (Agilent Technologies, USA).

### Cell culture

The human leukemic (U937) cell line was cultured in RPMI 1640. The human hepatocellular carcinoma (HepG2) cell line and normal African green monkey kidney epithelial (Vero) cell line were cultured in DMEM (GIBCO, Invitrogen Corporation, USA). Both media were supplemented with 10 % fetal bovine serum (GIBCO, Invitrogen Corporation), 100 units/mL penicillin and 100 µg/mL streptomycin (GIBCO, Invitrogen Corporation). The cells were cultured at 37 °C in a humidified atmosphere containing 5 % CO_2_.

### Antiproliferative effect

The antiproliferative activity of the *P. kesiya* extract in the leukemia (U937), hepatocellular carcinoma (HepG2), and normal (Vero) cell lines was assessed by neutral red assay [14, 15]. Briefly, 100 µL of U937 cells at a density of 5 × 10^5^ cells/mL and 100 µL of HepG2 or Vero cells at a density of 3 × 10^5^ cells/mL were independently seeded in 96-wells plates and incubated for 24 h. After cell growth, cells were treated with various concentrations (10 to 500 µg/mL) of the *P. kesiya* crude extract or melphalan (purity 95 %, Sigma–Aldrich Chemie GmbH, Germany) dissolved in DMSO (United States Biological, USA). The maximum final concentration of the compound was 500 µg/mL to maintain 1 % v/v DMSO with a cytotoxicity <10 % compared with the untreated cells. After treated cells were exposed to the test compound for 24 h, cells were stained directly with a final concentration of 50 µg/mL neutral red dye (Sigma Chemicals Co., USA) and incubated for another 2 h. The neutral red stained viable cells were dissolved by 0.33 % hydrogen chloride in isopropanol and detected using a colorimetric-based method. The absorbance was measured at 520 and 650 nm (reference wavelength) by a spectromicroplate reader (TECAN, Grödig, Austria). A plot of percentage cytotoxicity vs. test compound concentration was used to calculate the IC_50_.

### Apoptosis induction effect

#### Nuclei morphological study by 4´6-diamidino-2-phenylindole (DAPI) staining assay

The apoptosis effect of the plant crude extracts was primarily determined by fluorescent dye staining and DAPI to identify the condensation and fragmentation of nuclear DNA [14, 15]. Briefly, the cancer cells (HepG2 or U937) were treated with various concentrations of the test compounds (6 to 500 µg/mL of *P. kesiya* and 0.75 to 15 µg/mL of melphalan) for 24 h, after which the culture medium was removed and the cells were washed with fresh medium. Cells were then fixed by cold methanol. DAPI dye (Sigma–Aldrich Chemie GmbH, Germany) was then added to stain the nuclei DNA for 1 h. The excess dye was removed and 1X PBS (pH 7.4; 10 mM) was added to glycerin (at a 1:1 ratio). The DAPI staining assay was performed in triplicate in independent experiments. An inverted fluorescence microscope ​(Nikon eclipse 80i, Kanagawa, Japan) was used to record images of the DAPI staining. The average percentage of apoptotic cells was calculated from three independent wells with 10 eye views per well under the inverted fluorescence microscopy at a magnitude of 40×.

#### Analysis of DNA fragmentation

DNA fragmentation was analyzed [14, 15]. Briefly, after cancer cells were treated with various concentrations of *P. kesiya* extracts (60, 150, 300, 450, and 500 µg/mL) and melphalan (15 µg/mL) for 24 h, they were collected and washed with fresh medium. Then, the cell suspension was transferred to microcentrifuge tubes and centrifuged (Daihan Scientific, Seoul, Korea) at 300×*g* for 5 min to collect the cell pellet. The DNA in the cell pellet was extracted using a FlexiGene DNA kit (QIAGEN GmbH, Germany) then 2 µg of the DNA was analyzed using electrophoresis on 2 % agarose gels (Bio-Rad, USA) containing 0.1 mg/mL ethidium bromide (AppliChem, USA). DNA was mixed with loading dye (SibEnzyme Ltd., Russia) and the gel electrophoresed in 0.5X Tris–Borate-EDTA buffer (pH 8.3; 40 mMTris base, 45 mM boric acid, 1 mM EDTA; Sigma–Aldrich Chemie GmbH, Germany) at 250 V for 1 min and 20 V for 4 h. After electrophoresis, the DNA fragments were analyzed by In Genius L gel documentation (Syngene, USA).

### Enhancement of anticancer effect of *P. kesiya* extract in vitro

The enhanced chemosensitivity of melphalan combined with *P. kesiya* on the U937 and HepG2 cell lines was determined as per Chou and Talalay [22]. The antiproliferation assay on the melphalan and *P. kesiya* extract combination treatment against the HepG2 cancer cell line after 24 h was performed using the same method as for melphalan or *P. kesiya* extract alone. The cells were treated with the *P. kesiya* extract and melphalan was added subsequently. Different concentrations of the *P. kesiya* extract and melphalan combination treatment were used (Table 1). Briefly, the melphalan concentration was fixed at 1 × IC_50_ and the *P. kesiya* concentration was varied at 0.02, 0.05, 0.1, 0.2, 0.5, 1, 1.5, and 2 × IC_50_ in the U937 and HepG2 cell lines. In another series, the *P. kesiya* extract concentration was fixed at 1 × IC_50_ and the melphalan concentration was varied at 0.05, 0.1, 0.2, 0.5, 1, 1.5, and 2 × IC_50_ in both cancer cell lines. The maximum concentration that could be used in the experiment to maintain the percentage DMSO at less than 1 % v/v was 500 µg/mL, instead of a 2 × IC_50_ concentration of *P. kesiya* (600 µg/mL), the concentration used in this study was only 500 µg/mL. After 24 h exposure, the percentage of antiproliferation for each treatment was calculated.

**Table 1:** Concentration ratios of *P. kesiya* extract and melphalan combinations used in the combination anticancer study

IC_50_ Ratio in U937														
IC_50_ of *P. kesiya* (µg/mL)	0.02 (6)	0.05 (15)	0.1 (30)	0.2 (60)	0.5 (150)	1 (300)	1.5 (450)	2 (500^a^)	1 (300)	1 (300)	1 (300)	1 (300)	1 (300)	1 (300)
IC_50_ of melphalan (µg/mL)	1 (15)	1 (15)	1 (15)	1 (15)	1 (15)	1 (15)	1 (15)	1 (15)	0.05 (0.75)	0.1 (1.5)	0.2 (3)	0.5 (7.5)	1.5 (22.5)	2 (30)
IC_50_ Ratio in HepG2														
IC_50_ of *P. kesiya* (µg/mL)	0.02 (1.1)	0.05 (2.75)	0.1 (5.5)	0.2 (11)	0.5 (27.5)	1 (55)	1.5 (83)	2 (110)	1 (55)	1 (55)	1 (55)	1 (55)	1 (55)	1 (55)
IC_50_ of melphalan (µg/mL)	1 (40)	1 (40)	1 (40)	1 (40)	1 (40)	1 (40)	1 (40)	1 (40)	0.05 (2)	0.1 (4)	0.2 (8)	0.5 (20)	1.5 (60)	2 (80)

The experimental IC_50_ concentrations of *P. kesiya* in U937 and HepG2 cells were 299.0 ± 5.2 and 52.0 ± 5.8 µg/mL, respectively

The experimental IC_50_ concentrations of melphalan in U937 and HepG2 cells were 15.0 ± 1.0 and 37.7 ± 9.8 µg/mL, respectively

^a^The maximum concentration used in the experiment to keep percentage DMSO at <1 % v/v was 500 µg/mL

The combined effects were analyzed to determine whether the enhanced growth inhibitory effect was antagonistic, additive, or synergistic. The combination index (CI) was calculated (adapted from the multiple-drug effect analysis based on the median effect principle and the isobologram technique developed by Chou [22] and Eid et al. [23]). The CI took into account both the potency and shape of the dose–effect curve, which is given by Eq. (1):

1 $${\text{CI}} = \frac{{({\text{D}})_{1} }}{{({\text{D}}_{\text{X}} )_{1} }} + \frac{{({\text{D}})_{2} }}{{({\text{D}}_{\text{X}} )_{2} }}$$

CI > 1, = 1, and <1 correspond to synergism, addition, and antagonism, respectively. The denominators (D_x_)_1_ and (D_x_)_2_ are the doses of a single treatment of the first and second compound designated as **1** and **2**, respectively. Additionally, (D)_1_ and (D)_2_ are the doses of the compounds **1** and **2** in the combination treatment that exhibited x % antiproliferation.

The calculated dose reduction index (DRI) was used to determine the extent of dose reduction in the combination treatment compared with the dose of each single treatment. For the combination effect, DRI was defined as ${\text{DRI}}_{1} = {{({\text{D}}_{\text{X}} )_{1} } \mathord{\left/ {\vphantom {{({\text{D}}_{\text{X}} )_{1} } {({\text{D}})}}} \right. \kern-0pt} {({\text{D}})}}_{1} \,{\text{and}}\,{\text{DRI}}_{2} = {{({\text{D}}_{\text{X}} )_{2} } \mathord{\left/ {\vphantom {{({\text{D}}_{\text{X}} )_{2} } {({\text{D}})_{2} }}} \right. \kern-0pt} {({\text{D}})_{2} }}$. The relationship between DRI and CI was represented as: ${\text{CI}} = {1 \mathord{\left/ {\vphantom {1 {{\text{DRI}}_{1} }}} \right. \kern-0pt} {{\text{DRI}}_{1} }} + {1 \mathord{\left/ {\vphantom {1 {{\text{DRI}}_{2} }}} \right. \kern-0pt} {{\text{DRI}}_{2} }}$.

The same antiproliferation experiment was performed on the normal Vero cells to provide a comparison to determine the cytotoxic effect of combining the compounds against the normal cells. The synergistic apoptotic effects of the combination of *P. kesiya* extract and melphalan were evaluated against the U937 and HepG2 cells by DAPI staining and DNA fragmentation assay.

### Statistical analysis

Experiments were performed in triplicate and the results were expressed as a mean ± standard deviation ​from three independent experiments. Statistical analysis was performed by IBM SPSS Statistics 17.0 (SPSS Inc., Chicago, IL, USA). A two-way analysis of variance (ANOVA) test followed by Fisher’s least significant difference test for multiple comparisons of independent experiments were used to determine the effective enhancement of the inhibition growth by *P. kesiya* and melphalan in various cell lines. The significance level was set at *P* < 0.05. Statistical differences of percentage apoptotic cells between the *P. kesiya* treated group and the positive control group were compared by one-way ANOVA followed by Tukey’s honest significant difference test with a significance level of *P* < 0.05.

## Results

### Antiproliferative effects of *P. kesiya* extract and melphalan singly versus in combination on U937 and HepG2 cell lines

The antiproliferative effects of *P. kesiya* extract and melphalan singly or in combination were investigated in U937 and HepG2 cell lines. The IC_50_ values of *P. kesiya* in U937 and HepG2 were 299.0 ± 5.2 µg/mL and 52.0 ± 5.8 µg/mL, respectively. The IC_50_ values of melphalan in U937 and HepG2 were 15.0 ± 1.0 µg/mL and 37.7 ± 9.8 µg/mL, respectively. Both melphalan and *P. kesiya* extract caused significantly greater antiproliferation than was observed in untreated cancer cells (control). *P. kesiya* extract exerted potent antiproliferation in the HepG2 cells at an IC_50_ value of 52.0 ± 5.8 µg/mL. *P. kesiya* extract had a stronger selectivity against the two cancer cell lines than melphalan. A significant enhanced antiproliferative effect occurred with the combination when treating human U937 and HepG2 cell lines (Table 2). A two-way ANOVA was conducted that examined the effect of IC_50_ ratio and cell types on percentage antiproliferation. There was a statistically significant interaction between the effects of IC_50_ ratio and cell types on percentage antiproliferation, *F*(26, 84) = 42.654, *P* < 0.001. A two-way ANOVA detected significant differences in the antiproliferative activity of the various IC_50_ ratios (all *P* < 0.001) and among the various cell lines (*P* < 0.001). Significantly higher antiproliferative activities were observed at *P. kesiya* to melphalan concentration ratios of 0.2:1, 0.5:1, 1:1, 1.5:1, 2:1, 1:0.2, 1:0.5, 1:1.5, and 1:2 compared with normal Vero cells (*P* < 0.001). When the concentration of melphalan was constant, an enhanced antiproliferative effect was observed at *P. kesiya* to melphalan concentration ratios of 0.05:1 and 0.5:1 in the U937 and HepG2 cell lines, respectively. Alternatively, when *P. kesiya* was constant, the enhanced antiproliferative effect was observed at melphalan to *P. kesiya* concentration ratios of 0.1:1 and 0.2:1 in the U937 and HepG2 cell lines, respectively.

**Table 2:** Antiproliferation of *P. kesiya* extract and melphalan combination in U937, HepG2, and Vero cell lines

IC_50_ Ratio		% Antiproliferation		
*P. kesiya*	Melphalan	U937	HepG2	Vero
0	1	50	50	50^a^
1	0	50	50	Inactive^b^
1	1	100 ± 7.0	95.7 ± 12.2	6.8 ± 1.9
0.02	1	46.0 ± 5.7	Nd	Nd
0.05	1	59.8 ± 5.3	Nd	Nd
0.1	1	76.6 ± 13.5	Nd	1.0 ± 3.5
0.2	1	79.3 ± 3.7	43.7 ± 2.9	4.0 ± 6.1
0.5	1	100 ± 7.0	82.1 ± 4.9	7.3 ± 1.9
1.5	1	100 ± 2.2	93.4 ± 1.6	16.4 ± 4.7
2	1	100 ± 11.3	100 ± 3.1	24.7 ± 9.1
1	0.05	46.8 ± 9.5	34.2 ± 4.4	Nd
1	0.1	51.8 ± 5.5	46.0 ± 9.1	Nd
1	0.2	73.0 ± 4.2	60.7 ± 16.4	0 ± 3.3
1	0.5	76.4 ± 3.6	65.1 ± 9.1	1.8 ± 1.2
1	1.5	100 ± 2.0	100 ± 10.5	8.6 ± 1.4
1	2	100 ± 3.4	100 ± 4.9	9.5 ± 1.9

Two-way ANOVA detected significant antiproliferative activity differences among various IC_50_ ratios (*P* < 0.001) and among various cell lines (*P* < 0.001). A significant two-way interaction was observed between IC_50_ ratio and various cell lines (*P* < 0.001)

*Nd* not determined

^a^IC_50_ value of melphalan in Vero cells equals 59.9 ± 3.2 µg/mL

^b^Inactive when IC_50_ of *P. kesiya* in Vero cells was >500 µg/mL

Melphalan alone exhibited strong antiproliferation in normal Vero cells at an IC_50_ value of 59.9 ± 3.2 µg/mL, while *P. kesiya* extract alone was inactive in normal Vero cells. When treating the Vero cells, the antiproliferation of the combination treatment was <25 % when the concentration of melphalan was constant (Table 2). The result showed an antagonistic effect of *P. kesiya* extract on the antiproliferation of melphalan in Vero cells. In the presence of a 1 × IC_50_ concentration of *P. kesiya,* the concentration of melphalan used could be as high as 2 × IC_50_, producing only 9.5 ± 1.9 % antiproliferation against Vero cells.

### Synergistic effect of melphalan and *P. kesiya* extract combination analyzed in U937 leukemic and HepG2 hepatocellular carcinoma cell lines

**Fig. 1:**
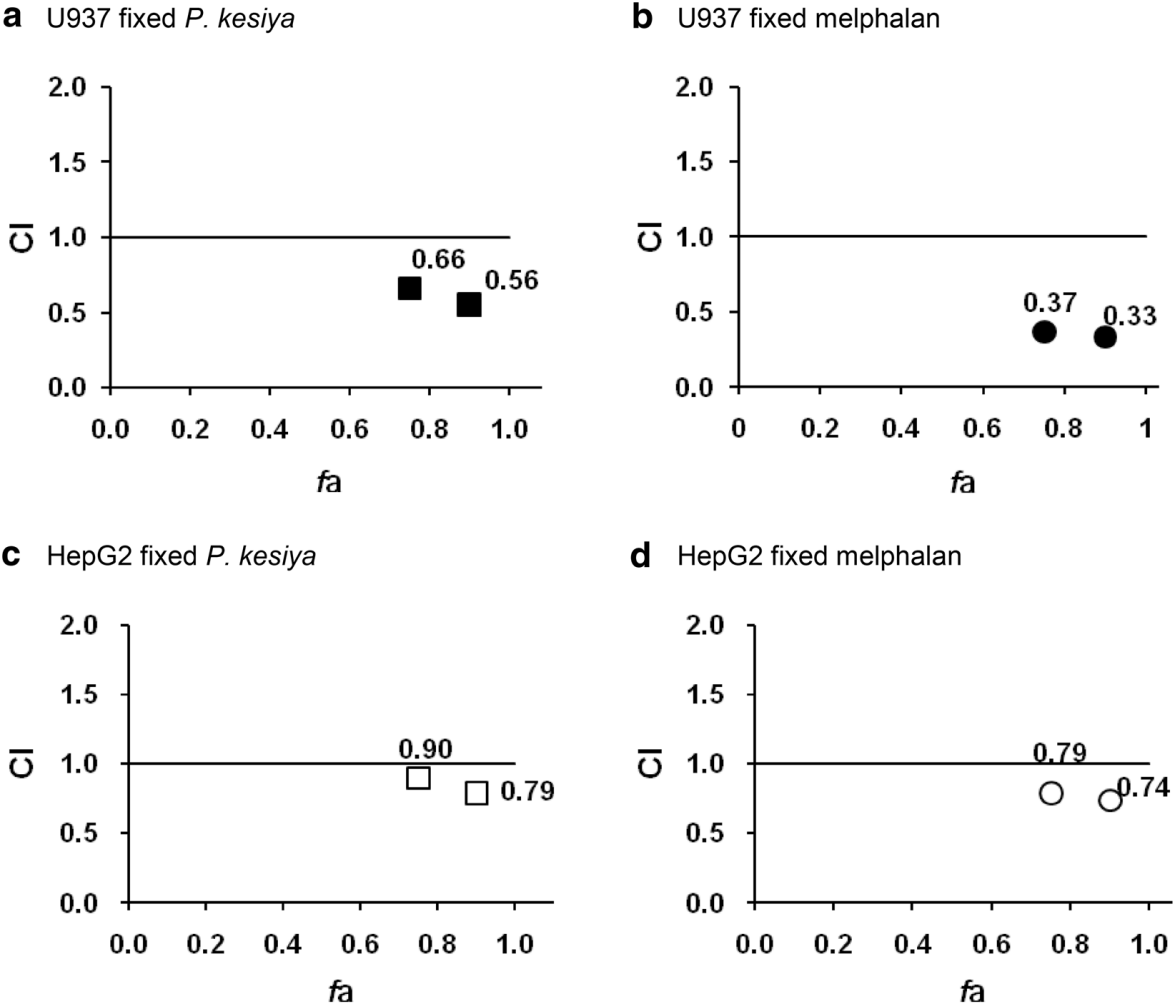
Isobolograms of the plot between combination index (CI) and *f*a in U937 (*black symbol*) and HepG2 (*white symbol*). *f*a = fraction affected by D (i.e., percentage antiproliferation/100). Isobolograms of the combination of compounds when *P. kesiya* was fixed (**a**, **c**; *square*), and when melphalan was fixed (**b**, **d**; *circle*) in each cancer cell lines

The CIs of the combined effect of *P. kesiya* and melphalan in the U937 and HepG2 cell lines at IC_75_ and IC_90_ values are presented in Table 3 and Fig. 1. Our results indicated that the combination of *P. kesiya* and melphalan exhibited high synergistic effects in U937 and HepG2 cells at concentrations producing 75 and 90 % antiproliferation (IC_75_ and IC_90_). As observed from the higher synergistic effect (low CI value), the combination therapy was more effective on the U937 cells than on the HepG2 cells.

**Table 3:** Dose reduction index (DRI) and combination index (CI)

% Antiproliferation	Cell lines	Combination treatment	DRI		CI
			*P. kesiya*	Melphalan	
90 % (IC_90_)	U937	Fixed [*P. kesiya*] = IC_50_	2.3	7.8	0.56
		Fixed [melphalan] = IC_50_	6.5	5.5	0.33
	HepG2	Fixed [*P. kesiya*] = IC_50_	2.0	3.6	0.79
		Fixed [melphalan] = IC_50_	2.4	3.2	0.74
75 % (IC_75_)	U937	Fixed [*P. kesiya*] = IC_50_	1.8	8.7	0.66
		Fixed [melphalan] = IC_50_	9.1	3.8	0.37
	HepG2	Fixed [*P. kesiya*] = IC_50_	1.6	3.8	0.90
		Fixed [melphalan] = IC_50_	2.7	2.4	0.79

DRI and CI of the combination treatment of *P. kesiya*/melphalan in U937 or in HepG2 cells at the concentrations that produced 90 and 75 % antiproliferation

### Extent of dose reduction in the combination treatment compared with single doses of each treatment

Table 3 shows the decreased concentration of melphalan or *P. kesiya* extract in the combination therapy to produce 90 and 75 % antiproliferation in U937 cells and HepG2 cells. The concentration of melphalan (when combined with a fixed concentration of *P. kesiya* extract) necessary to inhibit cancer growth by 90 % (IC_90_) represents a 7.8- and 3.6-fold decrease in U937 and HepG2 cells, respectively. By comparison, the concentration of *P. kesiya* extract (when combined with a fixed concentration of melphalan) needed for the IC_90_ represents a 6.5- and 2.4-fold decrease in U937 and HepG2 cells, respectively. A similar DRI trend (i.e., a greater melphalan dose reduction) for U937 cells compared with HepG2 cells was observed at 75 % antiproliferation. Dose reduction also depended on the type of cancer cells: 90 and 75 % cell death occurred when the *P. kesiya* extract concentration was fixed. The doses for melphalan per dose of *P. kesiya* extract could be reduced in the U937 cells (10.55:300 = 0.04:1 and 6.6:300 = 0.02:1) more than in the HepG2 cells (33.7:55.0 = 0.61:1 and 23.7:55.0 = 0.43:1). The synergistic effect appears to be greater for the melphalan because a greater potential dose reduction could be achieved; thus, melphalan concentrations could be significantly reduced.

### Apoptotic induction effect of single treatments and of *P. kesiya* extract and melphalan combination treatment

**Fig. 2:**
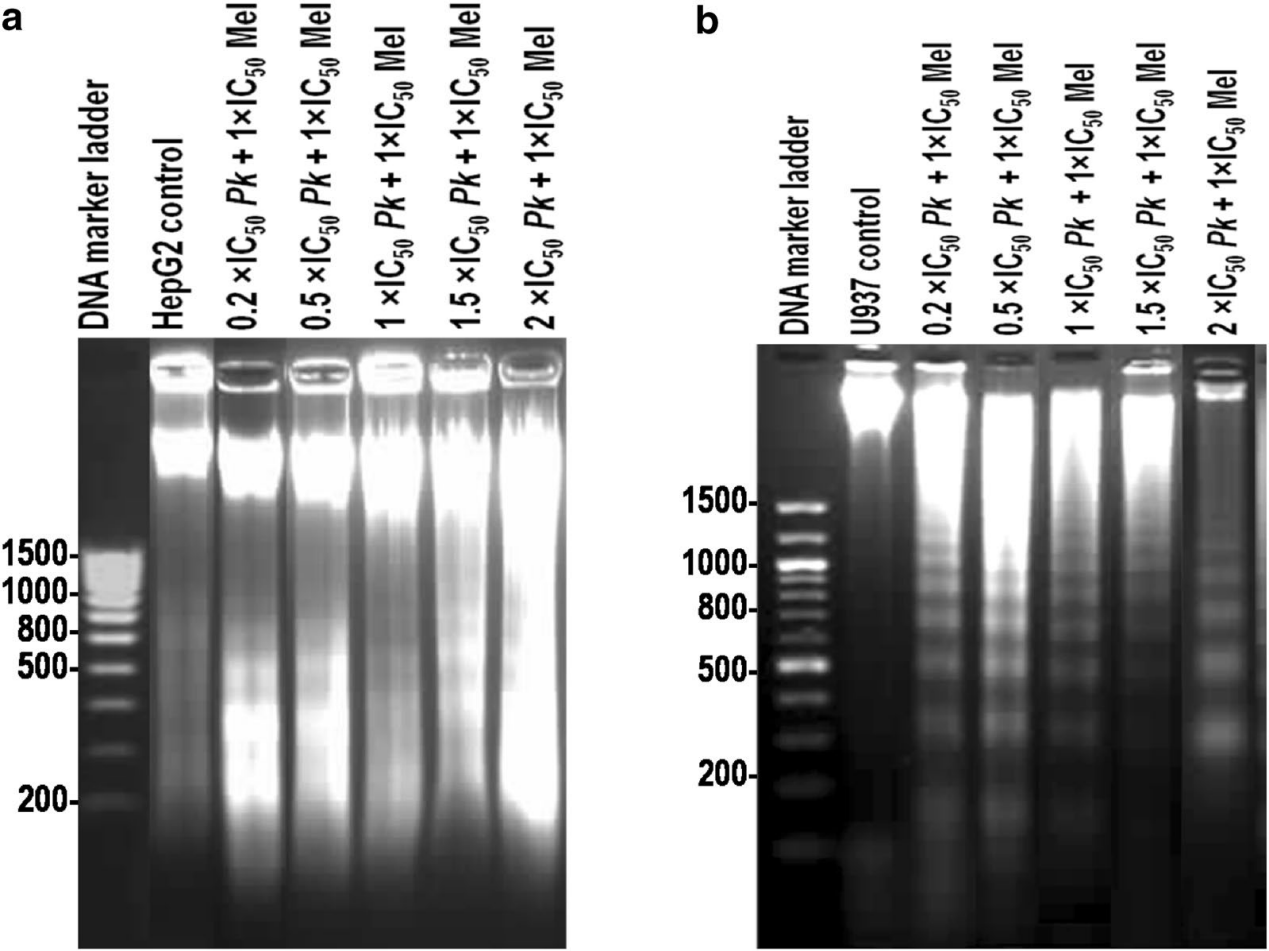
DNA fragments after combination treatment of *P. kesiya* (Pk) extract with melphalan in HepG2 (**a**) and U937 (**b**) cell lines

Apoptosis induction by *P. kesiya* extract and melphalan when used singly or in combination was evaluated in HepG2 and U937 cell lines using a method based on the DAPI staining assay (Table 4) and DNA fragmentation assays (Fig. 2). In the control group, the nuclei were roundish and homogeneously stained by DAPI; in contrast, the apoptotic nuclei in the treated U937 and HepG2 cells were irregularly shaped, small, detached, and had apoptotic bodies. At a 1 × IC_50_ concentration, *P. kesiya* extract induced 42.5 ± 4.8 and 39.7 ± 2.6 % apoptosis in the U937 and HepG2 cells, respectively; melphalan induced 43.1 ± 16.3 and 53.0 ± 6.1 % apoptosis, respectively. The combination therapy of *P. kesiya* extract and melphalan was significantly more effective in inducing apoptosis against U937 and HepG2 cells.

**Table 4:** Effects of *P. kesiya* and melphalan on apoptosis induction in U937 and HepG2 cells

IC_50_ ratio		% Apoptotic cells	
IC_50_ of *P. kesiya*	IC_50_ of Melphalan	U937	HepG2
0	1	43.1 ± 16.3	53.0 ± 6.1
1	0	42.5 ± 4.8^b^	39.7 ± 2.6^a^
1	1	100^a^	100^a^
0.1	1	77.0 ± 1.9^a^	66.7 ± 3.1^a^
0.2	1	100^a^	74.9 ± 12.3^a^
0.5	1	100^a^	92.8 ± 1.3^a^
1.5	1	100^a^	98.3 ± 3.0^a^
2	1	100^a^	100^a^
1	0.05	38.6 ± 6.1^a^	62.4 ± 3.2^a^
1	0.1	48.4 ± 3.8^a^	87.9 ± 3.3^a^
1	0.2	61.3 ± 3.3^a^	90.3 ± 6.0^a^
1	0.5	100^a^	100^a^
1	1.5	100^a^	100^a^
1	2	100^a^	100^a^

One-way ANOVA of percentage apoptotic cells compared with single treatment by melphalan at 1 × IC_50_ in cancer cells

^a^Significant difference (*P* < 0.001)

^b^Non-significant difference (*P* ≥ 0.001)

DNA fragmentation was determined according to whether the synergistic apoptosis induction in both cancer cells occurred toward the late stage of apoptosis, as previously observed for single therapy [15]. The combined treatment of *P. kesiya* with melphalan resulted in DNA laddering in both the HepG2 and U937 cells compared with the control cells (Fig. 2).

### GC–MS analysis of *P. kesiya* extract

GC–MS analysis of *P. kesiya* extract was performed to obtain its characteristic fingerprint (Table 5). The GC peaks obtained were used for further chemical constituent identification and revealed several compounds:podocarpa-8,11,13-trien-15-oic acid, neopine, rosin acid, pimaric acid, oleic acid, pyrocatecol, and vanillin (Table 5).

**Table 5:** Identified compounds and their relative distribution in *P. kesiya*

Retention time (min)	% of Total area	Assigned compounds
11.074	2.48	Pyrocatecol (1,2-benzenediol or brenzcatechin)
16.412	2.66	trans-Sobrerol
16.703	1.88	Vanillin
29.551	1.55	Palmitic acid
32.878	4.49	Oleic acid
35.650	4.16	Benzoyl isocyanate
36.096	4.77	Pimaric acid
37.170	6.52	Rosin acid
37.907	20.16	Podocarpa-8,11,13-trien-15-oic acid, 13-isopropyl
38.525	4.31	2,3-dimethoxy-5-[2-(3-hydroxy-4-methoxyphenyl)ethenyl]phenol or Combretastatin A3
41.788	5.98	Neopine

GC–MS data of the 50 % ethanol–water crude extract of *P. kesiya*

## Discussion

To our knowledge, this is the first report of a synergistic effect of *P. kesiya* extract on melphalan anticancer activity via the apoptosis induction mechanism in vitro. The U937 cells were only sensitive to melphalan, based on the lower IC_50_ value; however, the HepG2 cells were sensitive to both *P. kesiya* extract and melphalan. A wide chemopreventive index was observed in the combination treatment, probably because the cytotoxic effect was lower in the normal Vero cells. *P. kesiya* extract’s potent antiproliferation effect in the HepG2 cell line and high selective antiproliferation effect in both cancer cell lines requires further investigation.

Drug and herb combinations could significantly decrease the toxicity and chemoresistance of drugs and increase their efficacy [24, 25] i.e., increasing the efficacy of the therapeutic effect; decreasing the dosage to avoid toxicity; reducing the development of drug resistance; and providing selective synergism against a target (or efficacy synergism) vs. host (or toxicity antagonism). The ability of herbal extracts to enhance anticancer activity might also arise from the potentiation of pharmacokinetics, wherein one ingredient enhanced the therapeutic effect of another (active ingredient or drug) by modulating its pharmacokinetic properties (i.e., absorption, distribution, metabolism, and/or excretion) [24, 25]. The administration of multiple therapeutic agents is often associated with additional toxicities, which can be life threatening; however, a combination treatment with a non-toxic herbal extract like *P. kesiya* might provide superior benefits.

Previous work indicated that, in addition to the compounds identified here in *P. kesiya* extract (Table 5), gallic acid, chlorogenic acid, caffeic acid, vanillin, and coumaric acid in the 50 % ethanol–water extract of *P. kesiya* exhibited an anticancer effect [14]. The main compounds found in various parts of Pinaceae were terpenes, such as α- and β-pinene [26]. Rosin is composed of a complex mixture of different compounds, including resin acids such as abietic acid, plicatic acid, and pimaric acid. Resin acids might be derived from terpenes through partial oxidation and undergo isomerization in the presence of strong acids or with heat [27]. Hence, terpenes such as α- and β-pinene might undergo thermal conversion into resin acids under the GC–MS condition studied. One previous study reported that α-pinene expressed both apoptosis induction and antimetastatic activity against melanoma cells [26]. A previous work has also shown that citronellol—found in the family Pinaceae—inhibited an efflux P-gp protein at an IC_50_ value of 504 µM [28].

## Conclusions

Combining the *P. kesiya* extract with melphalan reduced toxicity while retaining the therapeutic efficacy (reduced approximately 4–9 fold) of melphalan.

## Abbreviations

DMEM: Dulbecco’s modified Eagle’s medium
DMSO: dimethylsulfoxide
FBS: fetal bovine serum
HepG2: human hepatocellular carcinoma cell line
NR: neutral red
PBS: phosphate buffer solution
NCI: National Cancer Institute (USA)
U937: human leukemic cell line
Vero: normal African green monkey kidney epithelial cell line
